# MIH and Cavities as Markers of Oral Health Inequality in Children from Southwest Andalusia (Spain)

**DOI:** 10.3390/dj13080345

**Published:** 2025-07-26

**Authors:** Leidy Bech Barcaz, David Ribas-Pérez, Paloma Villalva Hernandez-Franch, Luis El Khoury-Moreno, Julio Torrejón-Martínez, Antonio Castaño-Séiquer

**Affiliations:** Faculty of Dentistry, University of Seville, 41005 Seville, Spainpvillalva@us.es (P.V.H.-F.); lelkhoury@us.es (L.E.K.-M.); jtorrejon@us.es (J.T.-M.); acastano@us.es (A.C.-S.)

**Keywords:** dental caries, molar–incisor hypomineralisation (MIH), oral health, health inequalities, social determinants, migration and oral health

## Abstract

**Introduction:** Dental caries and molar–incisor hypomineralisation (MIH) are prevalent conditions affecting children’s oral health, with functional, aesthetic, and psychosocial implications. In Spain, previous studies have highlighted geographic and sociodemographic disparities in their distribution, particularly among rural and migrant populations. **Objective:** To characterise oral health status, in terms of caries and MIH, among 6–7-year-old children from the towns of Palos de la Frontera, Mazagón, and San Bartolomé. **Methods:** A cross-sectional study was conducted involving 229 children recruited from public primary schools. Sociodemographic, anthropometric, and behavioural data were collected through clinical examination and interview. Statistical analysis included univariate and multivariate logistic regression. The study protocol was approved by the Ethics Committee of Huelva. **Results:** The prevalence of caries (DMFT ≥ 1) was 53.3%, with mean DMFT and dft indices of 1.78 and 0.31, respectively. MIH affected 32.8% of the cohort, with a predominance in the first permanent molars (teeth 36 and 26). Multivariate analysis identified independent predictors of caries: African (OR = 7.47; 95% CI: 2.84–23.8) and European (OR = 4.56; 95% CI: 1.26–22.3) parental origin, poor oral hygiene (OR = 3.07; 95% CI: 1.60–6.03), and the presence of MIH (OR = 3.20; 95% CI: 1.64–6.42). The municipality of San Bartolomé was associated with a higher risk of MIH (OR = 2.90; 95% CI: 1.21–7.45). **Conclusions:** The high prevalence of caries and MIH in the Condado-Campiña district, exceeding national averages, reflects oral health inequities linked to social determinants (migrant origin, locality) and clinical factors (MIH, oral hygiene). Targeted preventive interventions are urgently needed in high-risk populations, including culturally tailored education and policies ensuring equitable access to dental care services.

## 1. Introduction

Oral health in childhood is vital to overall well-being, influencing physical growth, emotional well-being, social development, academic performance, and long-term quality of life. Early oral health issues can lead to both immediate and lasting negative effects across these areas [1,2]. Among the most prevalent oral conditions in paediatric populations is dental caries, which remains the most common oral disease worldwide, affecting approximately 520 million children and adolescents, with an average annual increase of 6.7% since 2010 [3]. In Spain, the prevalence among children aged 5 to 15 years is approximately 20.6%, with variability depending on geographic and social factors [4].

Dental caries is a dynamic, non-communicable, and multifactorial disease mediated by biofilms and modulated by diet, which results in net mineral loss from hard tissues and the formation of lesions [5]. Its onset and progression depend on biological, behavioural, psychosocial, and environmental factors, with high sugar intake, poor oral hygiene, insufficient fluoride and nutrient intake, and limited access to preventive services being key contributors [6,7]. These factors, together with socioeconomic, cultural, and geographic determinants [8], create a complex context that hinders the control of the disease despite advances in preventive and therapeutic strategies. Dental caries can cause pain, infection, and impaired chewing, affecting nutrition, development, and academic performance. They also impact psychosocial well-being, leading to low self-esteem and reduced oral health-related quality of life (OHRQoL) [9,10,11].

Molar–incisor hypomineralisation (MIH) has become a major concern in both research and clinical settings over the past 20 years. This is due to its high prevalence and harmful effects on children’s oral health. MIH is a qualitative defect in enamel. It mainly affects the first permanent molars and often the permanent incisors. Signs include clear opacities, enamel breakdown, and increased tooth sensitivity [12,13]. Recent studies [12] have reported a global average prevalence of 13.5% (95% CI: 12.0–15.1%), although figures vary widely by region, reaching 18.2% in Rome (Italy) and up to 39.9% in Syria [14,15]. In Spain, studies suggest a prevalence ranging from 7% to 25% in children aged 6 to 14 years, depending on the geographic and social context [16,17].

MIH is directly associated with increased susceptibility to caries, particularly in severe cases, and can result in additional complications such as chronic dental pain, intense hypersensitivity, and greater need for complex dental treatments. These issues significantly worsen children’s oral quality of life and increase both the emotional and financial burden on families and healthcare systems [18]. Although its aetiology is not yet fully understood, it is hypothesised that certain factors—such as adverse health events during the first five years of life and maternal illness during pregnancy—may play a critical role in its development [19].

This study focuses on children aged 6–7, a key stage for assessing caries and MIH due to the presence of both deciduous and newly erupted permanent molars. Evaluating this mixed dentition period supports early diagnosis, timely prevention, and analysis of the relationship between caries and MIH within relevant sociodemographic and behavioural contexts.

The aim of this cross-sectional study is to characterise oral health status, in terms of dental caries and MIH, among children aged 6–7 years located in the Condado-Campiña district. The study also seeks to identify sociodemographic, anthropometric, and behavioural factors associated with the occurrence of caries and MIH in this paediatric cohort. The findings will also contribute to the ongoing scientific debate regarding the aetiological factors underlying MIH by providing specific contextual data that complement and expand upon previous geographically and methodologically limited studies [20].

## 2. Materials and Methods

### 2.1. Study Design and Setting

An observational, cross-sectional, and analytical study was conducted in three municipalities in southwestern Spain: Palos de la Frontera, Mazagón, and San Bartolomé, all within the Condado-Campiña Health District.

Data collection activities included intraoral clinical examinations of schoolchildren, complemented by a brief clinical interview and anthropometric measurements (weight and height). The interview aimed to confirm parental nationality and assess oral hygiene practices.

Clinical examinations were carried out on school premises with logistical support from teaching staff. Each school provided a room that met basic requirements for privacy, appropriate lighting (both natural and artificial), and functional furnishings.

### 2.2. Participants

The study population consisted of children aged 6 to 7 years enrolled in state-funded primary schools (CEIP) in the selected localities. A simple random sampling method was applied using school enrolment records of eligible pupils. Children were invited to participate upon receipt of written informed consent from their parents or legal guardians.

Children with medical conditions that could hinder dental examination in the school setting without extraordinary accommodations were excluded, as were those whose parents or guardians explicitly refused participation. Given the non-invasive nature of the study protocol, no further exclusion criteria were applied, in order to maximise inclusivity and enhance the external validity of the findings.

### 2.3. Variables

#### 2.3.1. Dependent Variables

Dental caries: Caries presence was assessed through intraoral examination using a plain dental mirror, in accordance with World Health Organization (WHO) guidelines for oral health surveys [21]. Caries were recorded as a binary variable (present/absent), and the number of affected teeth was noted as a discrete quantitative variable, distinguishing between deciduous and permanent dentition.

Molar–Incisor Hypomineralisation (MIH): MIH was defined following the diagnostic criteria of the European Academy of Paediatric Dentistry (EAPD) [22].

#### 2.3.2. Independent Variables

Gender (binary nominal qualitative variable), Municipality (polytomous nominal qualitative variable), School, Oral hygiene (nominal qualitative variable), Weight (continuous quantitative variable), Height (continuous quantitative variable), Body mass index (BMI; continuous quantitative variable).

#### 2.3.3. Secondary Variables

dft index (decayed, filled teeth in deciduous dentition), DMFT index (in permanent dentition), Restoration index.

### 2.4. Data Sources

Data from intraoral examinations, interviews, and anthropometric assessments were first recorded in structured data collection forms and subsequently entered into a bespoke Microsoft Excel^®^2021 database developed for this study. All variables were systematically coded to ensure proper internal validation and facilitate data traceability during analysis.

Weight and height were measured by the school nurse and the paediatrician from the local health centre. Intraoral examinations were conducted tooth by tooth, with the permanent tooth recorded when it coexisted with a deciduous one. Permanent teeth were coded numerically, while primary teeth were denoted using letters in parentheses.

Once data collection was complete, all data were exported to R software version 4.5 for statistical analysis.

### 2.5. Sample Size

Sample size was calculated based on the development of a predictive model for the likelihood of dental caries or MIH occurrence, following the methodology described by Riley et al. [23]. The expected value of the Cox–Snell R^2^ for the new model was set at 0.3, with five candidate predictor variables and a shrinkage factor of 0.9 for internal validation.

Assuming an expected outcome proportion of 15%, the minimum required sample size was 196 participants. To account for an anticipated non-response rate of 10%, the minimum target sample was increased to 216 children.

### 2.6. Statistical Analysis

Quantitative variables were described using mean and standard deviation or median and interquartile range, depending on distribution. Qualitative variables were described using absolute and relative frequencies. Normality was assessed using the Shapiro–Wilk test.

To explore potential associations between independent variables (sex, municipality, BMI, oral hygiene) and dependent variables (caries, MIH), bivariate analyses were conducted. Appropriate statistical tests were selected based on variable type: chi-square or Fisher’s exact test for proportions; Student’s *t*-test, ANOVA, or non-parametric tests (Wilcoxon, Kruskal–Wallis) for continuous variables.

Variables identified as potential predictors were included in the multivariate analysis. Binary logistic regression models were used to estimate the independent effects of explanatory variables on the presence of dental caries and MIH, adjusting for potential confounders. Results were reported as adjusted odds ratios (OR) with corresponding 95% confidence intervals.

Collinearity was assessed using the variance inflation factor (VIF). All statistical tests were two-tailed, with a significance level set at *p* < 0.05. Statistical analyses were performed using R version 4.5 [24].

### 2.7. Ethical Considerations

The study was approved by the Research Ethics Committee of Huelva (SICEIA-2024-003240) and adhered to international ethical standards, the EU General Data Protection Regulation (GDPR 2016/679), and Spain’s Organic Law 3/2018 on data protection. Participation was voluntary, requiring written informed consent from parents or guardians, which included detailed information on study objectives and procedures.

## 3. Results

### 3.1. Participant Characteristics

As we can see in Table 1, a total of 229 children were included in the study, of whom 200 (87.3%) were aged 6 years and 29 (12.7%) were aged 7 years. There was a slight predominance of male participants (53.7%) compared to females (46.3%). The most represented municipality was Palos de la Frontera (54.6%), followed by San Bartolomé (26.2%) and Mazagón (19.2%). The majority of parents were of Spanish origin (73.8%), although a notable proportion had African backgrounds (17.0%). Regarding anthropometric variables, the mean weight of the children was 24.5 kg (SD = 5.7), and the mean height was 122.6 cm (SD = 4.7). The average body mass index (BMI) was 16.2 kg/m^2^ (SD = 3.0). The overall prevalence of overweight was 10.0%, while obesity was observed in 6.1% of the sample.

### 3.2. Prevalence of Caries and Associated Factors

**Figure 1:**
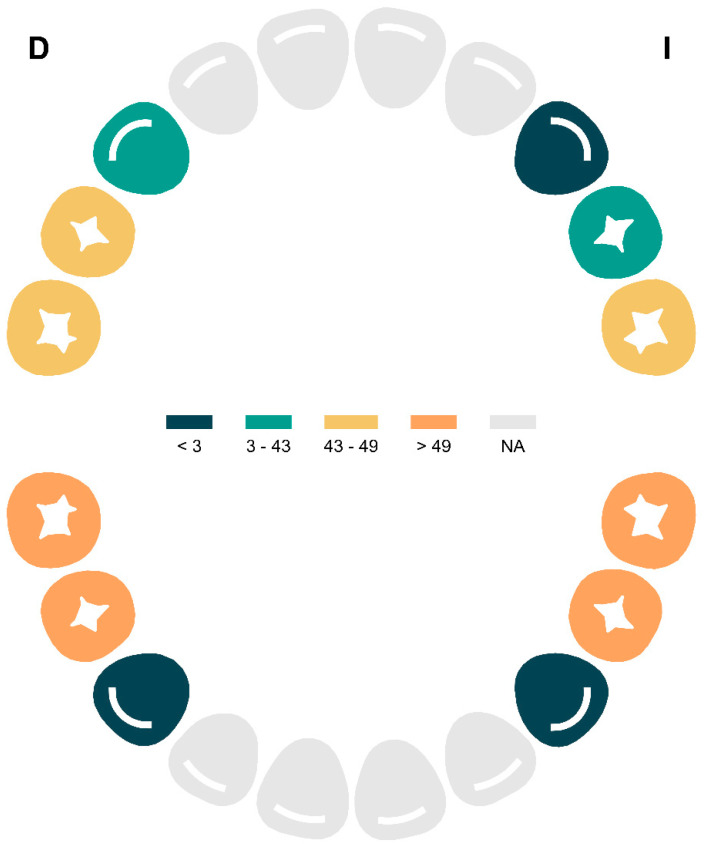
Anatomical Distribution of Caries Frequency in the Primary Dentition by Tooth. Abbreviations: NA = Not Affected.

The topographic analysis of caries distribution in the primary dentition revealed a predominant pattern of involvement in the posterior primary molars. The teeth with the highest frequency of carious lesions were 74 (51 lesions), 85 (50 lesions), 75 (49 lesions), and 84 (49 lesions), followed by teeth 55 (48 lesions), 54 (43 lesions), and 65 (43 lesions). In contrast, a lower frequency of caries was observed in the anterior teeth, with only 30 lesions in tooth 64, three in 53, two in 83, and one in 63, while tooth 73 showed no recorded lesions (Figure 1).

Regarding the permanent dentition, caries involvement was primarily concentrated in the first molars. The most affected teeth were tooth 26 (20 lesions), followed by teeth 36 and 46 (both with 15 lesions), and to a lesser extent, tooth 16 (12 lesions).

A total of 122 children (53.3%) presented with at least one carious lesion. Analysis of the association between participant characteristics and the presence of a DMFT index ≥ 1 revealed a statistically significant difference according to parental origin (*p* < 0.001). While 89.7% of children without caries had parents of Spanish origin, this proportion decreased to 59.8% among those with caries, with a notable increase in the proportion of parents of African origin (27.9% versus 4.7% in the caries-free group). No statistically significant differences were identified in sex distribution (*p* = 0.235), place of residence (*p* = 0.145), weight (*p* = 0.902), height (*p* = 0.429), or body mass index (BMI) (*p* = 0.997) between the groups with and without caries. Oral hygiene and molar incisor hypomineralisation were significantly associated with the presence of a DMFT index ≥ 1 (*p* < 0.001) (Table 2).

In the univariate analysis, significant associations were observed between dental caries and factors such as parental origin, oral hygiene, and the presence of MIH. Children with parents of African origin exhibited a markedly higher risk of caries (OR = 8.94; 95% CI: 3.62–27.1; *p* < 0.001), followed by those of European origin (OR = 5.70; 95% CI: 1.76–25.5; *p* = 0.008), compared to children with Spanish-origin parents. Similarly, poor oral hygiene (OR = 4.14; 95% CI: 2.30–7.64; *p* < 0.001) and MIH (OR = 3.56; 95% CI: 1.96–6.68; *p* < 0.001) showed a strong correlation with the presence of caries. The localities of Palos de la Frontera (OR = 1.72; *p* = 0.126) and San Bartolomé (OR = 2.17; *p* = 0.056) exhibited a tendency towards increased risk, although these findings were not statistically significant (Table 3).

In the multivariant analysis, African origin (OR = 7.47; 95% CI: 2.84–23.8; *p* < 0.001) and European origin (OR = 4.56; 95% CI: 1.26–22.3; *p* = 0.033) remained independent predictors of caries. Poor oral hygiene (OR = 3.07; 95% CI: 1.60–6.03; *p* < 0.001) and MIH (OR = 3.20; 95% CI: 1.64–6.42; *p* < 0.001) maintained their significant associations, reinforcing their role as key risk factors. Geographical differences between localities slightly attenuated their relevance (Palos de la Frontera: OR = 2.09; *p* = 0.079; San Bartolomé: OR = 2.46; *p* = 0.059), whereas sex showed no influence (*p* > 0.05) (Table 3).

### 3.3. Indices

The dft index was 1.78 (95% CI: 1.65–1.95), while the DMFT index was 0.31 (95% CI: 0.23–0.38) (Table 4). Based on the total number of decayed and filled primary and permanent teeth, the treatment need was estimated at 0.90 (95% CI: 0.87–0.93), whereas the restoration index was 0.1 (95% CI: 0.07–0.13).

### 3.4. Prevalence of MIH and Associated Factors

**Figure 2:**
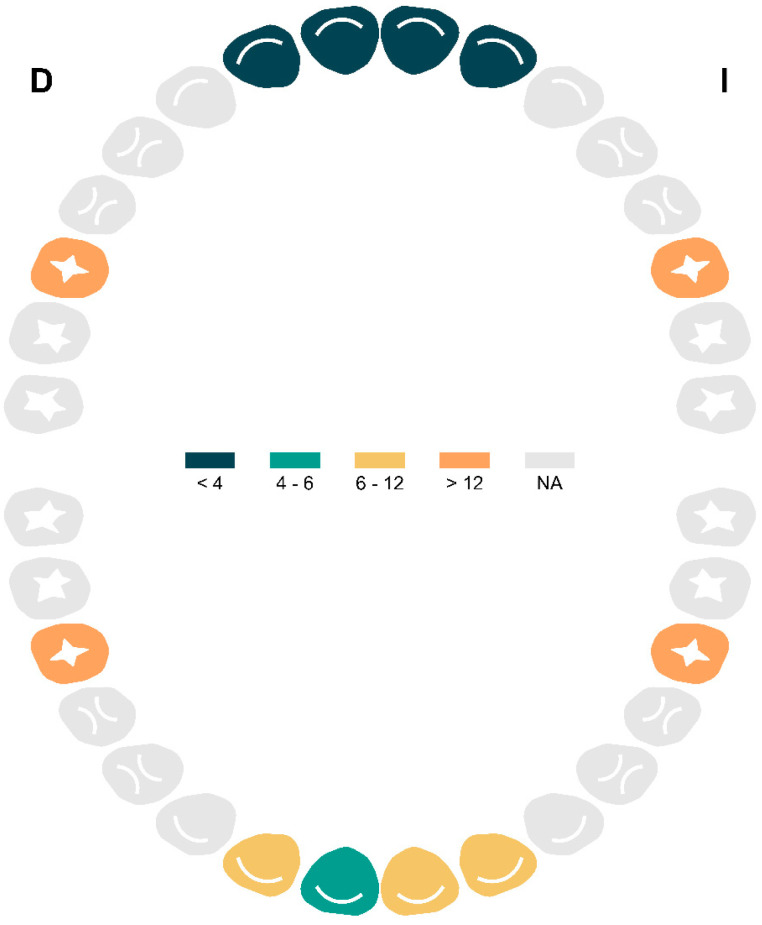
Anatomical distribution of the frequency of incisivo-molar hypomineralization in the permanent dentition by tooth. Abbreviations: NA = Not Affected.

The analysis of MIH frequency in the primary dentition showed that the posterior molars were the most commonly affected teeth. Tooth 85 presented the highest number of MIH cases, with 17 affected teeth, followed by tooth 75 with 11 cases, tooth 65 with nine cases, and tooth 55 with eight cases. Conversely, the analysis of permanent dentition revealed that the first permanent molars were the most frequently affected teeth. Tooth 36 showed the highest number of MIH cases, with 22 affected teeth, followed by tooth 26 with 18 cases, tooth 16 with 17 cases, and tooth 46 with 14 cases. Among the anterior teeth, the lower central and lateral incisors (31, 32, 41, 42) exhibited lower prevalence, with six, six, four, and six cases respectively. The upper incisors (11, 12, 21, 22) were the least affected, with frequencies of three, two, two, and two cases, respectively (Figure 2).

Table 5 presents the association analysis between sociodemographic and anthropometric characteristics and the presence of MIH. None of the variables analysed showed statistically significant differences between the groups with and without MIH (all *p*-values > 0.05). However, certain notable trends were observed: children with MIH exhibited a higher frequency of poor oral hygiene (76.4% vs. 64.3%; *p* = 0.069) and a higher prevalence were in San Bartolomé (34.7% vs. 22.3%; *p* = 0.066). Anthropometric variables (weight, height, and BMI) demonstrated similar distributions across both groups (*p* > 0.40). Parental origin and sex were also not significantly associated with the presence of MIH (*p* = 0.430 and *p* = 0.446, respectively).

The analysis of factors associated with MIH revealed that residents of San Bartolomé had a significantly higher risk in both the univariate analysis (OR = 2.78; 95% CI: 1.17–7.08; *p* = 0.025) and the adjusted multivariate analysis (OR = 2.90; 95% CI: 1.21–7.45; *p* = 0.021), compared with Mazagón (reference group). For Palos de la Frontera, a non-significant trend towards increased risk was observed (univariate OR = 1.70, *p* = 0.209; multivariate OR = 1.81, *p* = 0.161). Poor oral hygiene showed a marginally significant association in both models (univariate OR = 1.79, *p* = 0.071; multivariate OR = 1.85, *p* = 0.060) (Table 6).

## 4. Discussion

### 4.1. Main Findings

This study assessed oral health status in a paediatric population aged 6–7 years from the Condado-Campiña district, with a focus on dental caries and molar–incisor hypomineralisation (MIH). The findings revealed a caries prevalence of 53.3% (dft/DMFT ≥ 1) and a distinct pattern of involvement according to dentition type. In the primary dentition, the posterior molars—specifically teeth 74, 85, 75, and 84—exhibited the highest frequency of carious lesions. In contrast, in the permanent dentition, the first permanent molars (teeth 26, 36, and 46) were the most frequently affected. The dft index (1.78) and DMFT index (0.31) indicated a low burden of disease, accompanied by unmet treatment needs, as evidenced by the low restoration index (0.1).

Regarding factors associated with caries, parental origin emerged as a significant determinant of dental caries presence. Children with parents of African origin exhibited a substantially higher risk compared to those of Spanish origin (adjusted OR = 7.47; 95% CI: 2.84–23.8; *p* < 0.001). Similarly, children whose parents originated from other European countries—primarily Romania and Bulgaria, which comprised the majority of this subgroup—also showed a significantly increased risk (adjusted OR = 4.56; 95% CI: 1.26–22.3; *p* = 0.033). In addition to sociodemographic factors, poor oral hygiene (adjusted OR = 3.07; 95% CI: 1.60–6.03; *p* < 0.001) and the presence of MIH (adjusted OR = 3.20; 95% CI: 1.64–6.42; *p* < 0.001) were identified as independent predictors of caries.

With regard to MIH prevalence, 75 cases (32.8%) were recorded in the studied cohort, with the first permanent molars (teeth 36 and 26) being the most frequently affected. The locality of San Bartolomé showed a statistically significant association with MIH presence (OR = 2.90), while poor oral hygiene exhibited a borderline statistically significant trend (OR = 1.85; *p* = 0.060), suggesting a potential relationship warranting further investigation.

### 4.2. Comparison with the Literature

The findings of this study contrast with available national and regional data. At the national level in Spain, the 2020 Oral Health Survey reported a prevalence of DMFT > 0 of 28.3% among children aged 5–6 years, with significantly higher figures among populations of low socioeconomic status (47.3%) and children born abroad (59.8%) [4]. However, the prevalence observed in our study far exceeds the national average reported for Spanish children aged 2–10 years (21–25%, according to the 2020 Population Survey: Oral Health in Spain) [25]. This discrepancy may be explained by the sociodemographic characteristics of the study area, particularly the higher proportion of migrant families.

In the Andalusian context, our results align with previously reported intra-regional disparities. While the Fifth Epidemiological Survey of Oral Health in Schoolchildren in Andalusia [26] found a prevalence of 41.4% among 7-year-olds, local figures vary widely: from 21.7% in Granada to 53.19% in Lebrija and El Cuervo (Huelva) [20,27]. Our findings (53.3%) place the Condado-Campiña district at the upper end of this range, suggesting it may face similar challenges to those seen in Lebrija, such as pronounced socioeconomic inequalities or reduced coverage of oral health programmes. Furthermore, the restoration index of 10% in our cohort is markedly lower than the national average of 27.1% [4], highlighting a critical need for therapeutic interventions in this population.

Parental origin emerged as one of the strongest predictors of dental caries in this study, with particularly high risks among children of African (OR = 7.47) and other European origin (OR = 4.56). These findings are consistent with both national and international evidence identifying migrant status as a key determinant of oral health. In Spain, secondary analysis of the 2017 National Health Survey revealed that immigrant children were twice as likely to have caries, regardless of socioeconomic status [28]. Regional studies [29,30,31] support this trend, noting that immigrant children tend to exhibit higher levels of caries in both dentitions, with migrant status being the most influential variable in multivariate models.

Internationally, the prevalence of caries in primary dentition among immigrant children in Europe ranges from 22% to 88.7%, reflecting disparities linked to access to preventive care and socioeconomic conditions [32]. These wide ranges highlight the heterogeneity of challenges faced: while some groups encounter language or cultural barriers, others lack even basic health coverage. In our study, the magnitude of risk associated with African origin exceeds that reported in most of the literature, which may be attributable to unmeasured local factors such as high consumption of refined sugars or oral hygiene practices influenced by specific cultural norms [33,34].

Poor oral hygiene, identified in 68.1% of the study cohort (*n* = 156), emerged as a significant risk factor for dental caries (OR = 3.07; *p* < 0.001), reinforcing its central role in the pathogenesis of this disease. This finding is consistent with scientific evidence linking plaque accumulation—resulting from inadequate hygiene practices—with the proliferation of Streptococcus mutans and other acidogenic bacteria, the main contributors to enamel demineralisation and caries formation [1,35].

In sociodemographically diverse populations such as the one studied here, poor oral hygiene takes on added significance. Previous research has shown that migrant families, especially those in vulnerable socioeconomic conditions, often report lower frequencies of tooth brushing [36]. For instance, a study conducted in Melilla found that 41.6% of immigrant children brushed “rarely” [34]. This disparity may be due to cultural barriers (e.g., lower prioritisation of prevention) or a lack of tailored dental health guidance.

Molar–incisor hypomineralisation (MIH) was confirmed as an independent predictor of dental caries in this study (OR = 3.20; *p* < 0.001), a finding supported by consistent clinical and epidemiological evidence. Children with MIH show greater prevalence and severity of caries in the permanent dentition, particularly in affected first molars, where the risk of lesions extending into the dentine is up to four-times higher in severe MIH cases. This association is explained by the inherent structural weakness of hypomineralised enamel, which predisposes to post-eruptive breakdown and the formation of rough surfaces that favour bacterial biofilm adhesion and plaque accumulation. In vitro studies show that MIH-affected enamel contains 18–30% less mineral than healthy enamel, accelerating demineralisation in the face of acid attacks from cariogenic bacteria. In addition, hypersensitivity-related pain in MIH-affected teeth may hinder oral hygiene, perpetuating the cycle of deterioration [18,37,38,39].

MIH displays a heterogeneous distribution both globally and regionally, with prevalence affected by diagnostic criteria and environmental factors. A recent meta-analysis of 116 observational studies reported a global prevalence of 13.5% (95% CI: 12.0–15.1), with the highest rates in the Americas (15.3%) and the lowest in Asia (10.7%) [12]. In Spain, substantial regional differences are reported; a study in Madrid found a prevalence of 28.6% among children aged 8–11, with higher rates in girls (60.7%) and a predilection for upper first molars (74.3%) [40], while in Catalonia, the prevalence was significantly lower (12.2%), with no statistical differences between immigrant and native children [41]. Particularly noteworthy is the case of Lebrija and El Cuervo (Huelva), where the highest prevalence of MIH documented in Spain to date was recorded at 39.61%. These discrepancies may be explained by inconsistent application of the 2003 diagnostic criteria, as the global meta-analysis identified that lack of standardised case definitions increases result heterogeneity (*p* = 0.0066) [12].

In our findings, tooth number 36 was the most affected. There is no fixed pattern in MIH explaining why some teeth show higher frequency and severity than others. Prevalence can vary depending on the region, environmental factors, and diagnostic criteria used. However, since tooth 36 is one of the first permanent molars to erupt, it is often among the most evaluated and affected teeth in epidemiological studies of MIH [20,22,41,42,43].

As for risk factors, current evidence supports a multifactorial aetiology. Systematic reviews suggest weak but consistent associations with prenatal and perinatal events, such as maternal illness (e.g., infections), medication use during pregnancy, prematurity, and birth complications [42]. In early childhood, recurrent illnesses (e.g., fever, asthma, pneumonia) have been linked to an increased risk of MIH, possibly due to disruptions in amelogenesis during critical periods of dental development [43]. Additionally, the Catalonian study found that the presence of hypomineralisation in second primary molars (HSPM) increased the likelihood of developing MIH by 2.6 times, suggesting a shared biological predisposition [41].

While behavioural and socioeconomic factors—such as maternal alcohol consumption (associated with HSPM) or ethnic origin—have also been proposed, the validity of these associations is limited by methodological shortcomings, lack of confounder adjustment, and variability in exposure measurement [43]. This highlights the need for longitudinal studies with standardised protocols to clarify the aetiological mechanisms involved.

### 4.3. Strengths and Limitations

Among the main strengths of this study is its design, which employed representative sampling across three localities within the Condado-Campiña district, allowing for the capture of the region’s sociodemographic diversity. The use of standardised diagnostic criteria ensured the validity and reproducibility of the findings. Furthermore, the multivariate analysis adjusted for key variables such as oral hygiene, parental origin, and locality, thereby accurately identifying independent predictors of caries and MIH. The inclusion of detailed anthropometric and sociodemographic data enriched the cohort’s characterisation, providing a robust framework for interpreting the observed disparities.

However, the study also presents several limitations: Its cross-sectional design prevents the establishment of causal relationships or the assessment of the temporal progression of caries and MIH. The absence of data on dietary habits, fluoride exposure, and individual socioeconomic status limited the ability to explore additional risk factors. Lastly, although the sampling strategy was representative, it is possible that families experiencing extreme marginalisation did not have access to the schools where recruitment was conducted, thereby restricting the generalisability of the results. Future research should adopt longitudinal designs and incorporate environmental exposure biomarkers to further investigate the underlying aetiological mechanisms.

## 5. Conclusions

This study characterised the oral health status of 6–7-year-old children in the Condado-Campiña district, identifying a high prevalence of dental caries and molar–incisor hypomineralisation (MIH), both exceeding national and regional averages. The epidemiological profile revealed significant disparities, with children of African or European parental origin exhibiting a higher risk of caries, and MIH emerging as an independent predictor. Key determinants included poor oral hygiene, migrant parental origin, and place of residence, whereas anthropometric variables (weight, height, BMI) did not show significant influence. These findings underscore the need to implement targeted preventive programmes for high-risk populations, incorporating culturally tailored education, access to fissure sealants, and topical fluoride application, with particular emphasis on migrant communities and marginalised rural areas.

## Figures and Tables

**Table 1:** Sociodemographic and Anthropometric Characteristics of the Study Sample by Age Groups.

Variable	6 Years *n* = 200 ^1^	7 Years *n* = 29 ^1^	Total *n* = 229 ^1^
Gender			
Female	90 (45.0%)	16 (55.2%)	106 (46.3%)
Male	110 (55.0%)	13 (44.8%)	123 (53.7%)
Municipality			
Mazagón	40 (20.0%)	4 (13.8%)	44 (19.2%)
Palos de la Frontera	107 (53.5%)	18 (62.1%)	125 (54.6%)
San Bartolomé	53 (26.5%)	7 (24.1%)	60 (26.2%)
Parental Origin			
Spain	148 (74.0%)	21 (72.4%)	169 (73.8%)
Europe	14 (7.0%)	2 (6.9%)	16 (7.0%)
Africa	34 (17.0%)	5 (17.2%)	39 (17.0%)
America	4 (2.0%)	1 (3.4%)	5 (2.2%)
Weight (kg)			
Min–Max	15.7–67.5	20.1–33.0	15.7–67.5
Mean (SD)	24.5 (6.0)	24.1 (3.5)	24.5 (5.7)
Height (cm)			
Min–Max	105.0–138.0	118.0–139.0	105.0–139.0
Mean (SD)	122.6 (4.8)	122.9 (4.4)	122.6 (4.7)
BMI kg/m^2^			
Min–Max	11.8–37.0	13.0–20.2	11.8–37.0
Mean (SD)	16.2 (3.1)	16.0 (1.8)	16.2 (3.0)
Median (Q1–Q3)	15.5 (14.5–17.2)	16.0 (14.7–16.9)	15.6 (14.5–17.1)
Category BMI			
Severe Malnutrition	3 (1.5%)	1 (3.4%)	4 (1.7%)
Moderate Malnutrition	7 (3.5%)	1 (3.4%)	8 (3.5%)
Normal Weight	157 (78.5%)	23 (79.3%)	180 (78.6%)
Overweight	19 (9.5%)	4 (13.8%)	23 (10.0%)
Obesity	14 (7.0%)	0 (0.0%)	14 (6.1%)

^1^ *n* (%).

**Table 2:** Association between sociodemographic and anthropometric characteristics and the presence of dental caries.

Variable	Dft Index		*p*-Value ^2^
	Dft = 0 *n* = 107 ^1^	Dft ≥ 1 *n* = 122 ^1^	
Gender			0.235
Females	54 (50.5%)	52 (42.6%)	
Males	53 (49.5%)	70 (57.4%)	
Parental Origin			<0.001
Spain	96 (89.7%)	73 (59.8%)	
Europe	3 (2.8%)	13 (10.7%)	
Africa	5 (4.7%)	34 (27.9%)	
America	3 (2.8%)	2 (1.6%)	
Municipality			0.145
Mazagón	26 (24.3%)	18 (14.8%)	
Palos de la Frontera	57 (53.3%)	68 (55.7%)	
San Bartolomé	24 (22.4%)	36 (29.5%)	
Weight, kg	23.4 (21.0, 25.5)	23.0 (21.5, 26.0)	0.902
Height, cm	122.0 (119.5, 125.0)	121.0 (120.0, 124.5)	0.429
BMI, kg/m^2^	15.32 (14.55, 16.92)	15.66 (14.33, 17.24)	0.997
Oral Hygiene			<0.001
Good	51 (47.7%)	22 (18.0%)	
Poor	56 (52.3%)	100 (82.0%)	
MIH			<0.001
Healthy	88 (82.2%)	69 (56.6%)	
Present	19 (17.8%)	53 (43.4%)	

^1^ *n* (%); Median (Q1, Q3). ^2^ xi square test of independence; Fisher’s exact test; Wilcoxon Signed-Rank Test.

**Table 3:** Factors Associated with the Presence of Dental Caries: Results from Univariate and Multivariate Analyses.

Variable	Univariate Analysis			Multivariate Analysis		
	OR	95% CI	*p*-Value	OR	95% CI	*p*-Value
Gender						
Females	—	—		—	—	
Males	1.37	0.81, 2.32	0.235	1.29	0.70, 2.38	0.415
Parental Origin						
Spain	—	—		—	—	
Europe	5.70	1.76, 25.5	0.008	4.56	1.26, 22.3	0.033
Africa	8.94	3.62, 27.1	<0.001	7.47	2.84, 23.8	<0.001
America	0.88	0.11, 5.42	0.887	1.01	0.12, 7.49	0.992
Municipality						
Mazagón	—	—		—	—	
Palos de la Frontera	1.72	0.86, 3.50	0.126	2.09	0.93, 4.87	0.079
San Bartolomé	2.17	0.99, 4.85	0.056	2.46	0.98, 6.38	0.059
Oral Hygiene						
Good	—	—		—	—	
Poor	4.14	2.30, 7.64	<0.001	3.07	1.60, 6.03	<0.001
MIH						
Healthy	—	—		—	—	
Present	3.56	1.96, 6.68	<0.001	3.20	1.64, 6.42	<0.001

Abbreviations: CI = Confidence Interval, OR = Odds Ratio.

**Table 4:** Caries index in deciduous and permanent dentition.

	Decayed	Missing	Filled	Total (IC 95%)
dft	369	-	39	1.78 (1.65, 1.95)
DMFT	62	0	8	0.31 (0.23, 0.38)

Abbreviations: D = decayed, F = filled, M = missing.

**Table 5:** Association Between Sociodemographic and Anthropometric Characteristics and the Presence of Molar–Incisor Hypomineralisation.

	MIH		*p*-Value ^2^
	Healthy *n* = 157 ^1^	Present *n* = 72 ^1^	
Gender			0.446
Females	70 (44.6%)	36 (50.0%)	
Males	87 (55.4%)	36 (50.0%)	
Parental Origin			0.430
Spain	120 (76.4%)	49 (68.1%)	
Europe	9 (5.7%)	7 (9.7%)	
Africa	24 (15.3%)	15 (20.8%)	
America	4 (2.5%)	1 (1.4%)	
Municipality			0.066
Mazagón	35 (22.3%)	9 (12.5%)	
Palos de la Frontera	87 (55.4%)	38 (52.8%)	
San Bartolomé	35 (22.3%)	25 (34.7%)	
Weight, kg	23.2 (21.0, 26.7)	23.5 (22.1, 24.8)	0.424
Height, cm	121.0 (119.5, 125.0)	122.0 (120.0, 125.0)	0.404
BMI, kg/m^2^	15.35 (14.51, 17.16)	15.89 (14.48, 16.67)	0.633
Oral Hygiene			0.069
Good	56 (35.7%)	17 (23.6%)	
Poor	101 (64.3%)	55 (76.4%)	

^1^ *n* (%); Median (Q1, Q3). ^2^ xi square test of independence; Fisher´s exact test; Wilcoxon Signed-Rank Test.

**Table 6:** Factors Associated with the Presence of Molar–Incisor Hypomineralisation: Results from Univariate and Multivariate Analyses.

	Univariate Analysis			Multivariate Analysis		
	OR	95% CI	*p*-Value	OR	95% CI	*p*-Value
Province Municipality						
Mazagón	—	—		—	—	
Palos de la Frontera	1.70	0.77, 4.07	0.209	1.81	0.81, 4.38	0.161
San Bartolomé	2.78	1.17, 7.08	0.025	2.90	1.21, 7.45	0.021
Oral Hygiene						
Good	—	—		—	—	
Poor	1.79	0.97, 3.45	0.071	1.85	0.99, 3.60	0.060

Abbreviations: CI = Confidence Interval, OR = Odds Ratio.

## Data Availability

The raw data supporting the conclusions of this article will be made available by the authors on request.

## References

[1] Krol D.M., Whelan K., Section on Oral Health (2023). Maintaining and Improving the Oral Health of Young Children. Pediatrics.

[2] Watt S., Dyer T.A., Marshman Z., Jones K. (2024). Does poor oral health impact on young children’s development? A rapid review. Br. Dent. J..

[3] Guarnizo-Herreño C.C., Wehby G.L. (2012). Children’s dental health, school performance, and psychosocial well-being. J. Pediatr..

[4] Bravo Pérez M., Almerich Silla J., Canorea Díaz E., Casals Peidró E., Cortés Martinicorena F., Expósito Delgado A., Gómez Santos G., Hidalgo Olivares G., Lamas Oliveira M., Martínez Beneyto Y. (2020). Encuesta de Salud Oral en España 2020. Rev. Del Iluestre Cons. Gen. Col. De Odontólogos Y Estomatólogos España.

[5] Machiulskiene V., Campus G., Carvalho J.C., Dige I., Ekstrand K.R., Jablonski-Momeni A., Maltz M., Manton D.J., Martignon S., Martinez-Mier E.A. (2020). Terminology of Dental Caries and Dental Caries Management: Consensus Report of a Workshop Organized by ORCA and Cariology Research Group of IADR. Caries Res..

[6] Kirthiga M., Murugan M., Saikia A., Kirubakaran R. (2019). Risk Factors for Early Childhood Caries: A Systematic Review and Meta-Analysis of Case Control and Cohort Studies. Pediatr. Dent..

[7] Lam P.P.Y., Chua H., Ekambaram M., Lo E.C.M., Yiu C.K.Y. (2022). Risk predictors of early childhood caries increment-a systematic review and meta-analysis. J. Evid. Based Dent. Pract..

[8] Schwendicke F., Dörfer C.E., Schlattmann P., Foster Page L., Thomson W.M., Paris S. (2015). Socioeconomic inequality and caries: A systematic review and meta-analysis. J. Dent. Res..

[9] Ozsin Ozler C., Cocco P., Cakir B. (2020). Dental caries and quality of life among preschool children: A hospital-based nested case-control study. Br. Dent. J..

[10] Benelli K.D.R.G., Chaffee B.W., Kramer P.F., Knorst J.K., Ardenghi T.M., Feldens C.A. (2022). Pattern of caries lesions and oral health-related quality of life throughout early childhood: A birth cohort study. Eur. J. Oral Sci..

[11] Fernandez M.D.S., Pauli L.A., da Costa V.P.P., Azevedo M.S., Goettems M.L. (2022). Dental caries severity and oral health-related quality-of-life in Brazilian preschool children. Eur. J. Oral Sci..

[12] Lopes L.B., Machado V., Mascarenhas P., Mendes J.J., Botelho J. (2021). The prevalence of molar-incisor hypomineralization: A systematic review and meta-analysis. Sci. Rep..

[13] Almuallem Z., Busuttil-Naudi A. (2018). Molar incisor hypomineralisation (MIH)—An overview. Br. Dent. J..

[14] Nisii F., Mazur M., De Nuccio C., Martucci C., Spuntarelli M., Labozzetta S., Fratini A., Sozzi S., Maruotti A., Vozza I. (2022). Prevalence of molar incisor hypomineralization among school children in Rome, Italy. Sci. Rep..

[15] Al-Nerabieah Z., AlKhouli M., Dashash M. (2023). Prevalence and clinical characteristics of molar-incisor hypomineralization in Syrian children: A cross-sectional study. Sci. Rep..

[16] Garcia-Margarit M., Catalá-Pizarro M., Montiel-Company J.M., Almerich-Silla J.M. (2014). Epidemiologic study of molar-incisor hypomineralization in 8-year-old Spanish children. Int. J. Paediatr. Dent..

[17] Hernández M., Boj J.R., Espasa E., Peretz B. (2018). Prevalencija molarno-incizivne hipomineralizacije u skupini španjolske školske djece. Acta Stomatol. Croat. Int. J. Oral Sci. Dent. Med..

[18] Afzal S.H., Skaare A.B., Wigen T.I., Brusevold I.J. (2024). Molar-Incisor Hypomineralisation: Severity, caries and hypersensitivity. J. Dent..

[19] Juárez-López M.L.A., Salazar-Treto L.V., Hernández-Monjaraz B., Molina-Frechero N. (2023). Etiological Factors of Molar Incisor Hypomineralization: A Systematic Review and Meta-Analysis. Dent. J..

[20] Jiménez Moreno E. (2015). Salud Bucodental de la Cohorte de 6 años en Lebrija y El Cuervo: Prevalencia de la Hipoplasia Incisivo-Molar y Valoración del Grado de Satisfacción y Mejora de la Asistencia Dental Infantil.

[21] Petersen P.E., Baez R., World Health Organization (2013). Assessment of oral status. Oral Health Surveys Basic Methods.

[22] Lygidakis N.A., Garot E., Somani C., Taylor G.D., Rouas P., Wong F.S.L. (2022). Best clinical practice guidance for clinicians dealing with children presenting with molar-incisor-hypomineralisation (MIH): An updated European Academy of Paediatric Dentistry policy document. Eur. Arch. Paediatr. Dent..

[23] Riley R.D., Ensor J., Snell K.I.E., Harrell F.E., Martin G.P., Reitsma J.B., Moons K.G.M., Collins G., van Smeden M. (2020). Calculating the sample size required for developing a clinical prediction model. BMJ.

[24] R Core Team (2025). R: A Language and Environment for Statistical Computing.

[25] Blanco L., Llodra Calvo J.C. (2020). Encuesta Poblacional: La Salud Bucodental en España 2020.

[26] Bravo Pérez M., Cabrera León A., Llodra Calvo J.C. (2022). V Estudio Epidemiológico de la Salud Bucodental Escolar en Andalucía. https://www.juntadeandalucia.es/export/drupaljda/v_estudio_epidemiologico_salud_oral_escolares_andaluces.pdf.

[27] Monteagudo C., Téllez F., Heras-González L., Ibañez-Peinado D., Mariscal-Arcas M., Olea-Serrano F. (2015). Hábitos dietéticos de los escolares e incidencia de caries dental. Nutr. Hosp..

[28] Portero de la Cruz S., Cebrino J. (2020). Oral Health Problems and Utilization of Dental Services Among Spanish and Immigrant Children and Adolescents. Int. J. Environ. Res. Public. Health.

[29] Almerich-Silla J.M., Montiel-Company J.M. (2007). Influence of immigration and other factors on caries in 12- and 15-yr-old children. Eur. J. Oral Sci..

[30] Silla J.M.A., Montiel Company J.M. (2006). Oral health survey of the child population in the Valencia Region of Spain (2004). Med. Oral Patol. Oral Cir. Bucal.

[31] Ribas-Pérez D., Sevillano Garcés D., Rodriguez Menacho D., Hernandez-Franch P.V., Barbero Navarro I., Castaño Séiquer A. (2023). Cross-Sectional Study on Oral Health-Related Quality of Life Using OHIP-14 in Migrants Children in Melilla (Spain). Children.

[32] Banihashem Rad S.A., Esteves-Oliveira M., Maklennan A., Douglas G.V.A., Castiglia P., Campus G. (2024). Oral health inequalities in immigrant populations worldwide: A scoping review of dental caries and periodontal disease prevalence. BMC Public Health.

[33] Esteban-Gonzalo L., Veiga O.L., Gómez-Martínez S., Regidor E., Martínez D., Marcos A., Calle M.E. (2013). Adherence to dietary recommendations among Spanish and immigrant adolescents living in Spain; the AFINOS study. Nutr. Hosp..

[34] Kizi G., Raquel Barata A., Ventura I., Flores-Fraile J., Ribas-Perez D., Castaño-Seiquer A. (2023). Oral Health in migrants children in Melilla, Spain. Children.

[35] Wu Y., Li G., Lyu C.H., Zhou N., Wong H.M. (2024). Oral microbiota in preschoolers with rampant caries: A matched case-control study. Appl. Microbiol. Biotechnol..

[36] Skeie M.S., Riordan P.J., Klock K.S., Espelid I. (2006). Parental risk attitudes and caries-related behaviours among immigrant and western native children in Oslo. Community Dent. Oral Epidemiol..

[37] Oreano M.D., Santos P.S., Borgatto A.F., Bolan M., Cardoso M. (2023). Association between dental caries and molar-incisor hypomineralisation in first permanent molars: A hierarchical model. Community Dent. Oral Epidemiol..

[38] Gevert M.V., Wambier L.M., Ito L.Y., Feltrin de Souza J., Chibinski A.C.R. (2024). Which are the clinical consequences of Molar Incisor hypomineralization (MIH) in children and adolescents? Systematic review and meta-analysis. Clin. Oral Investig..

[39] Americano G.C., Jacobsen P.E., Soviero V.M., Haubek D. (2017). A systematic review on the association between molar incisor hypomineralization and dental caries. Int. J. Paediatr. Dent..

[40] Ortega-Luengo S., Feijóo-Garcia G., Miegimolle-Herrero M., Gallardo-López N.E., Caleya-Zambrano A.M. (2024). Prevalence and clinical presentation of molar incisor hypomineralisation among a population of children in the community of Madrid. BMC Oral Health.

[41] Cots E., Casas M., Gregoriano M., Busquet-Dura X., Bielsa J., Chacon C., Kragt L., Torán P., Guinot F. (2024). Ethnic disparities in the prevalence of Molar-Incisor-Hypomineralisation (MIH) and caries among 6–12-year-old children in Catalonia, Spain. Eur. J. Paediatr. Dent..

[42] Franco M.M.P., Ribeiro C.C.C., Ladeira L.L.C., Thomaz E.B.A.F., Alves C.M.C. (2024). Pre- and perinatal exposures associated with molar incisor hypomineralization: Birth cohort, Brazil. Oral Dis..

[43] Silva M.J., Scurrah K.J., Craig J.M., Manton D.J., Kilpatrick N. (2016). Etiology of molar incisor hypomineralization-A systematic review. Community Dent. Oral Epidemiol..

